# 

**Published:** 1909-02

**Authors:** 


### CONTENTS—FEBRUARY, 1909.—VOL. XXIV, NO. 8.

ORIGINAL ARTICLES—

The Ethical Past of the Man at the Helm.................... 319

Diabetes Mellitus. By J. C. Densten, M. D., Scranton, Pa...... 327

Acute Mastoiditis—Early Operation a Necessity for the Patient's Safety. By Robert E. Moss, M. D., San Antonio, Texas...... 333

EDITORIAL DEPARTMENT—

Song of the First Lord.................................. 337

Chronicles of the Octopus................................ 338

CORRESPONDENCE........................................ 348

SELECTED................................................ 354

BOOKS AND MAGAZINES.................................. 358

PUBLISHER'S DEPARTMENT.............................. 361

